# Prenatal diagnosis and postnatal follow-ups of 38 clubfoot cases in a tertiary reference center

**DOI:** 10.1590/1806-9282.20240938

**Published:** 2024-12-02

**Authors:** Refaettin Sahin, Atakan Tanacan, Hakki Serbetci, Osman Onur Ozkavak, Murat Haksever, Mehmet Utku Basarir, Ozgur Kara, Dilek Sahin

**Affiliations:** 1 Turkish Ministry of Health Ankara City Hospital, Department of Obstetrics and Gynecology, Division of Perinatology – Ankara, Turkey.; 2 University of Health Sciences, Turkish Ministry of Health Ankara City Hospital, Department of Obstetrics and Gynecology, Division of Perinatology – Ankara, Turkey.

**Keywords:** Clubfoot, Pregnancy, Ultrasound

## Abstract

**OBJECTIVE:**

The objective of this study was to evaluate the descriptive outcomes of 38 prenatally suspected clubfoot cases.

**METHODS:**

This is a retrospective cohort study conducted in Ankara Bilkent City Hospital's perinatology clinic. All consecutive cases with the diagnosis of fetal clubfoot between 2020 and 2023 were included. Multiple pregnancies and skeletal dysplasias were excluded from the study. Demographic features, prenatal ultrasound findings, prenatal screening test results, invasive diagnostic test results, clinical approaches, short-term and long-term postnatal outcomes, and treatment types of clubfoot were reported.

**RESULTS:**

There were a total of 38 prenatally diagnosed clubfoot cases noted during the study period. The mean gestational age at diagnosis was 15.3±1.4 weeks. Prenatal ultrasound screening noted bilateral clubfoot in 16 (42.1%) cases and unilateral clubfoot in 22 (57.8%) cases. Clubfoot was isolated in 7 (18.4%) cases, and additional anomalies were present in 31 (81.6%) cases. The gender of the fetuses was 26 (68.4%) males and 12 (31.6%) females. The prenatal noninvasive screening test was high risk in 7 (18.4%) patients and low risk in 16 (42.1%) patients. As a result of amniocentesis performed in 16 (42.1%) patients, abnormal karyotypes were detected in 2 patients (trisomy 18, 22q11.2 del). Six pregnancies were terminated before 24 weeks, and 27 pregnancies resulted in live birth at an average of 37.8±2.5 weeks. The diagnosis was correct in 84.2% of the cases, and 84.6% of the pregnancies resulted in live births.

**CONCLUSION:**

The outcomes of congenital clubfoot are generally favorable, especially in isolated cases.

## INTRODUCTION

Clubfoot (talipes equinovarus) is a developmental deformity of the foot and ankle, in which the foot is flexed plantarly with inward-facing sole. Ultrasound diagnosis can be made by acquiring both the plantar surface of the foot and lower extremity bones in the same sagittal plane. Some false positive diagnoses may be made due to the positional changes and uterine abnormalities. Clubfoot can be categorized into three groups: idiopathic, syndromic, and positional. Congenital clubfoot reported between 1 and 2 in 1,000 births^1^. The ultrasound diagnosis rate of clubfoot is reported as 60% with the median diagnosis being in the 18th-gestational week^2^. The detection rate is rising due to many factors such as improvements in ultrasound devices and improvements in physician education^3^. Although it is usually idiopathic, clubfoot deformity can be associated with other abnormalities such as trisomy 18, trisomy 21, trisomy 13 and 4p deletion, and other musculoskeletal and thoracic deformities^4^. In a systematic review that involved 5,458 cases, the ratio of isolated clubfoot was reported as 82%, and 11% of cases were reported as being associated with major congenital anomalies^5^. Clubfoot can be seen both unilaterally and bilaterally. The male-to-female ratio was reported as 2 to 1 in other studies^6^. The outcomes and postnatal treatment methods are closely associated with the severity of the deformity and coexisting abnormalities^7,8^. More and more congenital abnormalities can be detected prenatally due to the advances in imaging technology and improving knowledge of obstetricians. However, general knowledge regarding the outcomes of prenatally detected clubfoot cases is limited and more data are necessary to obtain more definitive results^9^.

The aim of this study was to evaluate the descriptive outcomes of 38 prenatally suspected clubfoot cases.

## METHODS

This retrospective cohort study was conducted on all consecutive prenatally detected clubfoot cases followed in Ankara Bilkent City Hospital's perinatology clinic between January 2020 and August 2023. The study protocol was approved by the ethics committee with the reference number E2-23-5054, and all participants gave written consent. Noninvasive screening results, invasive diagnosing results, demographic features, prenatal ultrasound findings, and postnatal outcomes were reported.

All ultrasound assessments were made with Voluson E10 with a 2–9-Mhz abdominal convex probe by the same expert perinatologist (DS). The diagnostic criterion was accepted as the plantar surface of the entire fetal foot and fibula and tibia being seen in the same plane. The first fetal ultrasound screening was performed at the 14th–16th week of gestation, and more ultrasound screenings were performed 2 weeks intervally until the time of delivery. Cases with a prenatal diagnosis of clubfoot were included. Multiple pregnancies and skeletal dysplasia were excluded from the study. All cases were evaluated postnatally by a pediatrician and an orthopedic specialist. Clubfoot was defined as the position of the hindfoot in equinovarus with the forefoot in adductus and cavus on physical examination^9^.

The statistical analysis was performed by SPSS 22 (IBM Corp., NY). The Kolmogrov-Smirnov test was used to assess whether the data are normally distributed. Mean and standard deviation values were used for normally distributed continuous variables, whereas median and range values were used to present continuous variables without normal distribution. Categorical variables were presented as numbers and percentages.

## RESULTS

There were a total of 38 prenatally diagnosed clubfoot cases noted during the study period. Demographic features and clinical approaches of all clubfoot cases are summarized in Table 1. Prenatal ultrasound screening noted bilateral clubfoot in 16 (42.1%) cases and unilateral clubfoot in 22 (57.8%) cases. Clubfoot was isolated in 7 (18.4%) cases, and additional anomalies were present in 31 (81.6%) cases. The prenatal noninvasive screening test was high risk in 7 (18.4%) patients and low risk in 16 (42.1%) patients. The karyotype results of 13 patients were reported as a normal karyotype, trisomy 18 for one patient, 22q:11.2 deletion (DiGeorge syndrome) for one patient, and maternal contamination for one patient. Six pregnancies were terminated before 24 weeks due to major congenital anomalies: Arnold-Chiari malformation (n=2), rhombencephalosynapsis and hydrocephalus (n=1), fetal akinesia deformation sequence (n=1), corpus callosum agenesis and hydrocephalus (n=1), and trisomy 18 with multiple congenital anomalies (n=1). One pregnancy resulted in abortus in the 16th week. Four pregnancies ended with fetal demise after the 20th week due to hydrops (n=2), massive bilateral pleural effusion (n=1), and complex cardiac anomaly (n=1). Short-term postnatal outcomes of clubfoot live birth cases are summarized in Table 1. Ten of the newborns were admitted to the neonatal intensive care unit. The reasons for admission to intensive care were as follows: hypotonia (n=1), DiGeorge syndrome (n=1), transposition of great arteries (n=1), hydrocephalus (n=1), ventricular septal defect (n=1), respiratory distress (n=2), omphalocele (n=1), and prematurity (n=2). In the postnatal examination of all 38 cases [missed abortus (n=1), in utero ex fetus (n=4), terminated fetuses (n=6), born alive (n=27)], correct diagnosis was present in 84.2% of the cases. It was confirmed that the diagnosis of clubfoot was not correct in 6 fetuses and that the diagnosis was correct in the remaining 32 fetuses. The long-term (one-year period) postnatal outcomes of live birth cases with clubfoot are summarized in Table 2. Twenty-seven live-born cases were followed up by a pediatrician, a developmental-behavioral pediatrician, and an orthopedist for one year. The baby diagnosed with DiGeorge syndrome died due to multiple infections after being monitored in the neonatal intensive care unit for 11 months. The correct diagnosis was made in 84.6% of the cases, and four babies’ feet were evaluated as normal. Of the 22 cases, 9 (40.9%) received serial casting followed by a surgical correction, 7 (31.8%) received the Ponseti (series of casting and orthotic bracing) treatment, and 6 (27.3%) received exercise and observation therapy.

**Table 1:** Demographic features, clinical approaches, and perinatal outcomes of all clubfoot cases n=38.

Maternal age		29.5±3.9*
Gravidity		2 (1–4)**
Parity		1 (0–3)**
Gestational age at diagnosis		15.3±1.4*
Noninvasive screening test	High risk	7 (18.4%)
	Low risk	16 (42.1%)
	Refused	15 (39.5%)
Amniocentesis^1^	Normal karyotype	13
	Trisomy 18	1
	DiGeorge syndrome	1
	Maternal contamination	1
Fetus gender	Male	26 (68.4%)
	Female	12 (31.6%)
Clubfoot side	Unilateral	22 (57.8%)
	Bilateral	16 (42.1%)
With additional anomalies	Yes	31 (81.6%)
	No	7 (18.4%)
Clinical approach	Missed abortus	1 (2.7%)
	Intrauterine fetal demise	4 (10.5%)
	Termination	6 (15.8%)
	Live births	27 (71%)
Correct diagnosis		32 (84.2%)
Birth week^2^		37.8±2.5*
Birth weight (gram)		2,882±629*
Apgar score 1st minute (range)		7 (4–8)**
Apgar 1st minute <7 (n, %)		5 (18%)
Apgar score 5th minute (range)		8 (5–10)**
Apgar 5th minute <7 (n, %)		4 (14%)
NICU admission***		10 (37%)

* Mean±standard deviation;

** Median, range (min–max);

1 Amniocentesis was indicated in 16 cases;

*** NICU: hypotonia (n=1), DiGeroge syndrome (n=1), great artery transposition (n=1), hydrocephalus (n=1), ventricular septal defect (n=1), respiratory distress (n=2), omphalocele (n=1), prematurity (n=2);

2 27 pregnancies resulted in livebirth. NICU: neonatal intensive care unit.

**Table 2:** Long-term (one-year period) postnatal outcomes of clubfoot live-birth cases n=27.

Survivors	Yes	26
	No	1*
Diagnosis	Correct	22 (84.6%)
	Misdiagnosis	4 (15.4%)
Treatment modality	Serial casting followed by surgical correction	9 (40.9%)
	Ponseti treatment	7 (31.8%)
	Exercise and observation	6 (27.3%)

* DiGeorge syndrome case, due to multiple infections.

## DISCUSSION

The findings of the present study indicated that the detection rate is relatively high, perinatal outcomes are generally favorable, and postnatal individualized treatment methods are mostly successful in prenatally diagnosed clubfoot cases.

Although there are various reports in the literature evaluating the perinatal outcomes of congenital clubfoot cases, the overall knowledge regarding the comprehensive outcomes is still limited. Postnatal outcomes were evaluated in a single-center retrospective study that included 109 clubfoot fetuses diagnosed prenatally. It was observed as an isolated finding in 76 (69.7%) of the fetuses and with additional anomalies in 33 (30.2%) of the fetuses. Amniocentesis was performed on 48 pregnant women, and chromosomal anomalies were detected in 6 (12.5%) of them. Postnatal diagnosis was confirmed in 65 (71.4%) of 91 live births. Postnatal confirmation of bilateral clubfoot was found to be higher than unilateral ones. The confirmation rate was found to be higher in singletons than in twins. Fetuses with bilateral clubfoot underwent further prenatal testing, but no increased frequency of additional anomalies was detected. The study concluded that the diagnosis rate in singleton pregnancies is higher than in multiple pregnancies, bilateral cases do not increase the frequency of additional anomalies, and karyotype analyses should be performed as if they were isolated^10^. Unlike our study, the association with additional anomalies was found to be higher, which may be due to the high number of cases referred from external centers. The frequency of chromosomal anomalies appeared to be similar 2/15 (13.3%), and the diagnosis rate in live births was higher 22/26 (84.6%) compared to the mentioned study.

In a single-center retrospective study including 65 patients, unilateral and bilateral clubfoot cases were evaluated. The false positivity rate was found to be 10.5%. It was concluded that 13% of babies followed up with isolated clubfoot were complex in the postnatal period. No chromosomal anomaly was detected in any baby during the neonatal period. 76.5% of 34 babies were followed with serial casting for 2 years. All children followed as isolated clubfoot had no walking problems. As a result of the study, it was concluded that false positivity was around 10%, and 10–13% of the cases followed as isolated clubfoot were actually complex^11^.

In a single-center retrospective study including 52 fetuses prenatally diagnosed with clubfoot, postnatal outcomes were evaluated. At the first examination, isolated clubfoot was detected in 40 (76.9%) of the fetuses, and complex clubfoot was detected in 12 (23.1%). During follow-up, the diagnosis was changed to indicate that 1 fetus was normal, 20 fetuses were complex, and 31 fetuses were isolated clubfoot. In the postnatal period, clubfoot diagnosis was confirmed in 40 fetuses, with a positive predictive value of 83%. Abnormal results were detected in 3 (12%) of 25 cases in which karyotype analysis was performed, and all of these cases were in the complex group^12^.

The findings of the present study were consistent with the previous literature. The prognosis is favorable, especially in isolated and unilateral cases. Although clubfoot may be associated with chromosomal abnormalities, the majority of the cases had a normal karyotype. Correct diagnosis can be achieved with targeted sonography. Postnatal prognosis seems good with necessary interventions.

The main strengths of the present study were its single-center experience and the relatively high number of study parameters. The main limitations were the retrospective design and lack of information regarding the further long-term prognosis of the cases.

In conclusion, further studies and data collection must be made to improve the diagnoses and evaluation of clubfoot cases. The outcomes of congenital clubfoot are generally favorable, especially in isolated cases.

## ETHICS APPROVAL

This study was approved by the Local Ethics Committee under approval number E2-23-5054.
